# Non disseminative nano-strategy against in vivo *Staphylococcus aureus* biofilms

**DOI:** 10.1038/s41522-023-00405-4

**Published:** 2023-06-17

**Authors:** Rita M. Pinto, Saleh Yazdani, Catarina Leal Seabra, Martine De Jonge, Mukaddes Izci, Rebeca Cruz, Susana Casal, Stefaan J. Soenen, Salette Reis, Cláudia Nunes, Patrick Van Dijck

**Affiliations:** 1 LAQV, REQUIMTE, Departamento de Ciências Químicas, Faculdade de Farmácia, Universidade do Porto, 4050-313 Porto, Portugal; 2 Laboratory of Molecular Cell Biology, Institute of Botany and Microbiology, KU Leuven, 3001 Leuven, Belgium; 3 NanoHealth and Optical Imaging Group, Translational Cell and Tissue Research Unit, Department of Imaging and Pathology, KU Leuven, Leuven, Belgium

**Keywords:** Biofilms, Pathogens, Antimicrobials, Biological techniques

## Abstract

*Staphylococcus aureus* is considered a high priority pathogen by the World Health Organization due to its high prevalence and the potential to form biofilms. Currently, the available treatments for *S. aureus* biofilm-associated infections do not target the extracellular polymeric substances (EPS) matrix. This matrix is a physical barrier to bactericidal agents, contributing to the increase of antimicrobial tolerance. The present work proposes the development of lipid nanoparticles encapsulating caspofungin (CAS) as a matrix-disruptive nanosystem. The nanoparticles were functionalized with D-amino acids to target the matrix. In a multi-target nano-strategy against *S. aureus* biofilms, CAS-loaded nanoparticles were combined with a moxifloxacin-loaded nanosystem, as an adjuvant to promote the EPS matrix disruption. In vitro and in vivo studies showed biofilm reduction after combining the two nanosystems. Besides, the combinatory therapy showed no signs of bacterial dissemination into vital organs of mice, while dissemination was observed for the treatment with the free compounds. Additionally, the in vivo biodistribution of the two nanosystems revealed their potential to reach and accumulate in the biofilm region, after intraperitoneal administration. Thus, this nano-strategy based on the encapsulation of matrix-disruptive and antibacterial agents is a promising approach to fight *S. aureus* biofilms.

## Introduction

*Staphylococcus aureus* is considered a high priority pathogen by the World Health Organization since it is a leading cause of nosocomial and community-acquired infections^1,2^. These bacteria are commonly found in infected implantable medical devices, such as catheters, and are responsible for high morbidity and mortality rates^3^. A virulent factor that highly contributes to the survival of *S. aureus* is the ability to form a biofilm. In these structures, the extracellular polymeric substances (EPS) matrix surrounds bacterial cells, protecting them from the harsh external environment and antibiotics^4^. The EPS matrix is a physical barrier to the diffusion of antimicrobial agents, leading to the failure of conventional therapies solely based on the administration of antibiotics^5^. Hence, the EPS matrix highly contributes to antimicrobial resistance phenomena. Novel therapeutic approaches that target not only bacterial cells within the biofilm but also the EPS matrix are critical to eradicate *S. aureus* biofilms.

Caspofungin (CAS) is an echinocandin antifungal agent used to treat infections caused by *Candida* spp. and *Aspergillus* spp. The mechanism of action of this fungicidal agent consists in inhibiting the synthesis of β-(1,3)-D-glucan, an essential component of the fungal cell wall^6^. However, this compound has been recently highlighted for its activity against bacterial biofilms^7,8^. Siala et al. (2016) reported that CAS inhibited the enzymatic activity of IcaA, which shares homology with fungal β-(1,3)-D-glucan synthases^8^. In *S. aureus*, the IcaA enzyme plays a critical role in the production of the EPS matrix component poly-*N*-acetylglucosamine (PNAG)^9^. Due to its matrix-disruptive properties, CAS revealed a potential combinatory effect with moxifloxacin (MOX) against *S. aureus* biofilms^8^. However, both CAS and MOX are prone to degradation, have a high affinity to plasma proteins, and present adverse side effects at high doses^10,11^. Hence, several factors may limit the drugs to reach the biofilm at therapeutic doses.

Nanotechnology is a promising field for drug delivery purposes, improving the efficacy of encapsulated drugs. Besides protecting encapsulated drugs, nanoparticles may also be designed to specifically target a site of interest^12,13^. Amongst the colloidal carriers described in the literature, lipid nanoparticles (LNPs) have been highlighted for their excellent biocompatibility and biodegradability. Besides, LNPs are highly stable, low cost, and easily functionalized to target a specific site^12^.

**Fig. 1:**
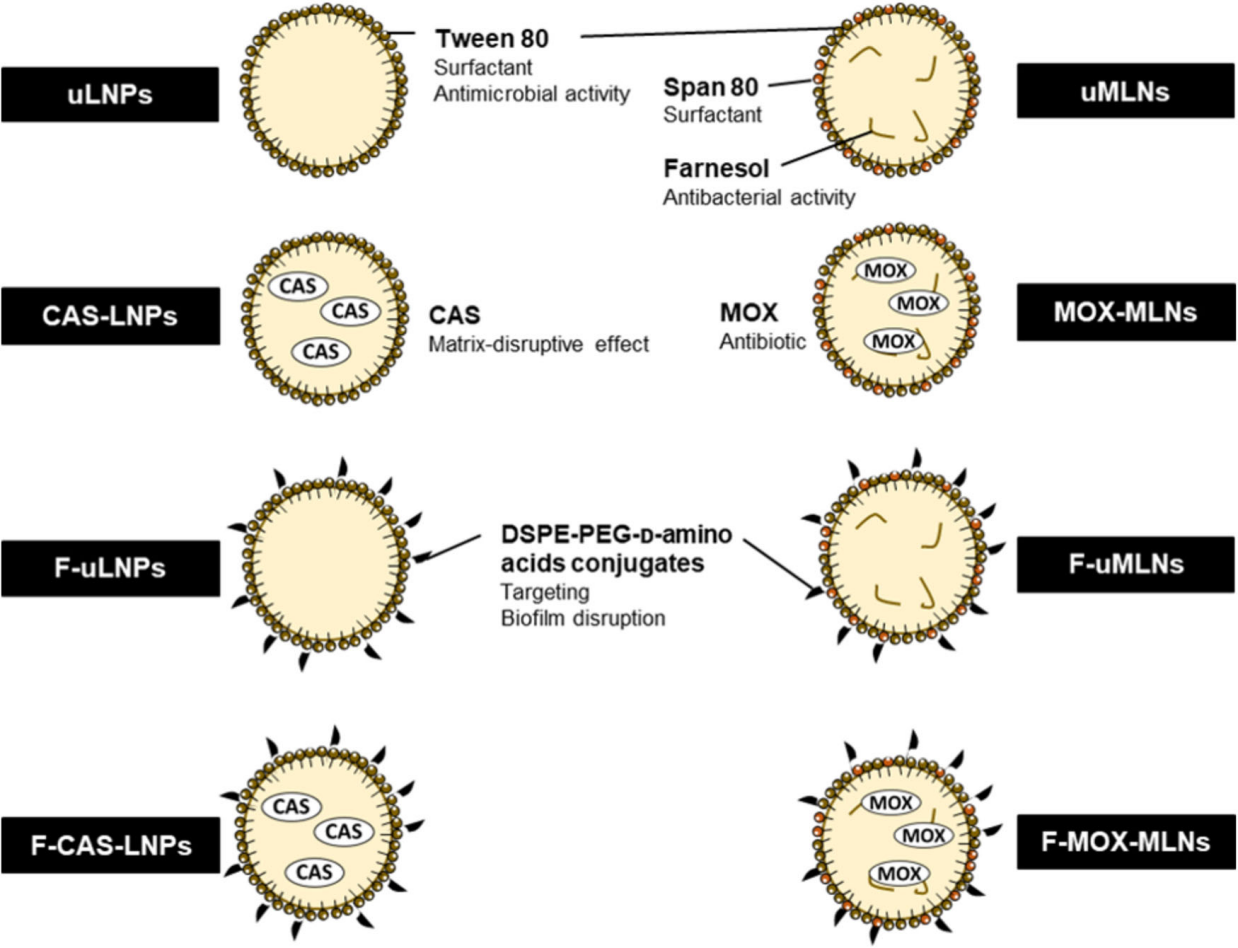
Schematic overview of the CAS-loaded and MOX-loaded nanosystems and respective unloaded formulations. CAS-loaded LNPs, composed of cetyl palmitate (solid lipid) and Tween^®^80, were produced by the double emulsion method^39^. The MOX-loaded multiple lipid nanoparticles (MLNs) were composed of cetyl palmitate, Miglyol^®^812 (liquid lipid), farnesol, and surfactants (Tween^®^80 and Span^®^80)^36^. The surface of the nanoparticles was functionalized with DSPE-PEG-D-amino acid conjugates (D-Phenylalanine, D-Proline, and D-Tyrosine). CAS, caspofungin; DSPE-PEG, 1,2-distearoyl-sn-glycero-3-phosphoethanolamine-N-[amino(polyethylene glycol)-2000] (ammonium salt); LNPs, lipid nanoparticles; MLNs, multiple lipid nanoparticles; MOX, moxifloxacin.

In this work, functionalized LNPs encapsulating CAS were developed and characterized in a novel approach towards the eradication of *S. aureus* biofilms. The nanosystems were functionalized with a mixture of D-amino acids (D-Phenylalanine, D-Proline, and D-Tyrosine) conjugated with polyethylene glycol (PEG). This mixture of D-amino acids aims to target and disrupt the biofilm specifically. PEG was added to the formulation to increase the circulation time of the LNPs, by avoiding recognition by the immune system^14,15^. The produced formulations were assessed in vitro for their cytocompatibility and antibiofilm efficacy. In a step forward towards an efficient antibiofilm treatment against *S. aureus* biofilms, CAS-loaded LNPs were combined with MOX-loaded nanosystems to evaluate potential combinatory effects, both in vitro and in vivo. Additionally, the in vivo biodistribution of these two nanosystems was assessed. In this multi-target strategy, encapsulated CAS (matrix-disruptive agent) was used as an adjuvant to improve the activity of MOX (bactericidal agent) against the bacterial biofilms. A schematic overview of the nanosystems used in this work is represented in Fig. 1.

## Results

### Physical characterization of LNPs and storage stability

**Fig. 2:**
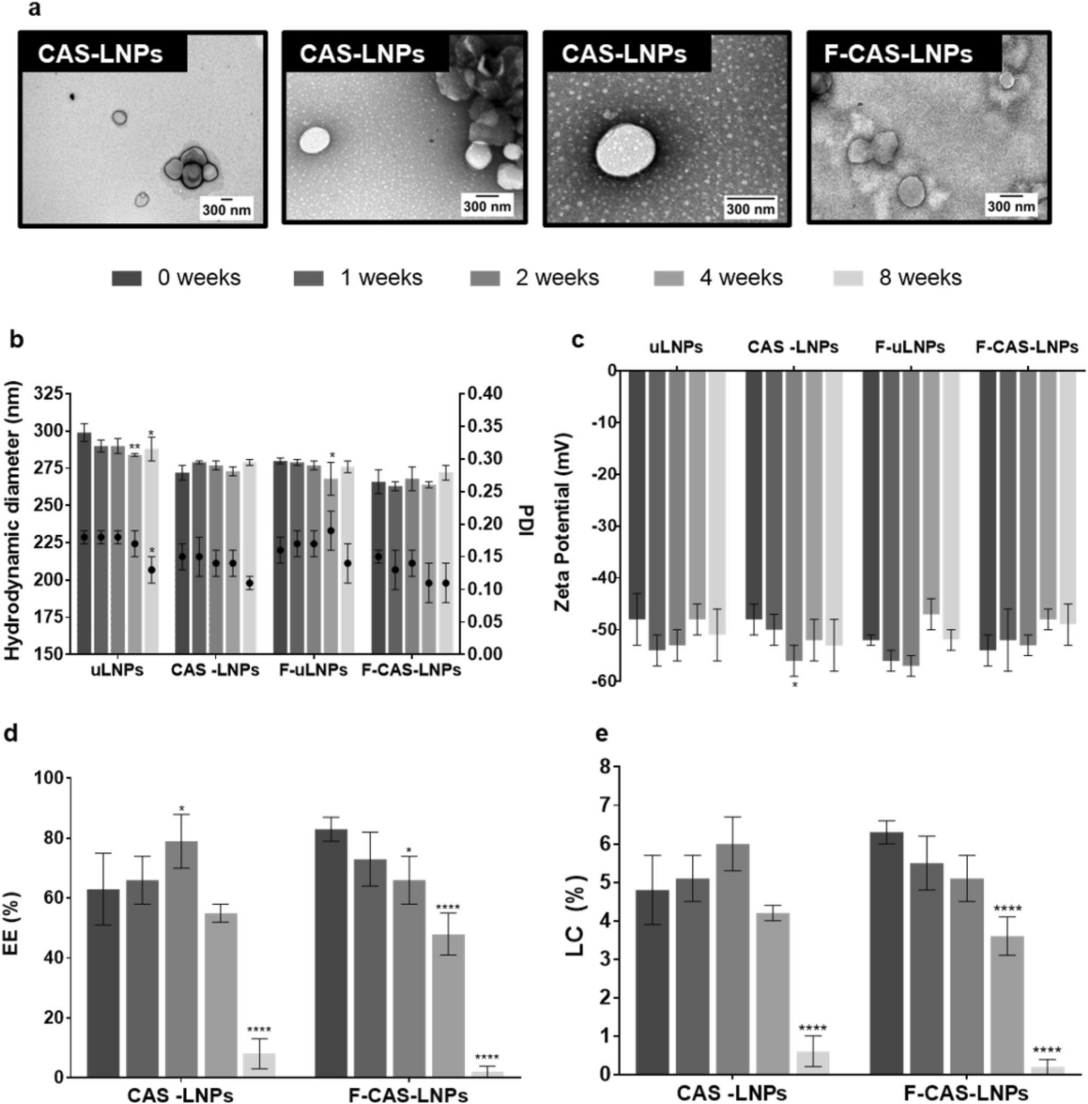
Physical characterization of the LNPs. **a** Transmission electron microscopy images of the CAS-LNPs and the F-CAS-LNPs. CAS-LNPs images were acquired at the magnifications of 25 000× (left), 50 000× (middle), and 100 000× (right), while F-CAS-LNPs were imaged at 50 000×. **b**–**e** Characterization of the LNPs suspensions over 8 weeks, stored at 4 °C. **b** Hydrodynamic diameter and PDI. The bars represent the hydrodynamic diameter (left y-axis) and the dots represent the PDI (right y-axis). **c** Zeta potential. **d** Encapsulation efficiency. **e** Loading capacity. The parameters were evaluated at different time points (0, 1, 2, 4, and 8 weeks). The values are represented as the mean ± SD. *p < 0.05, **p < 0.01, ****p < 0.0001, relatively to 0 weeks. Statistical analysis: two-way ANOVA, Dunnett’s multiple comparisons test.

The produced LNPs were characterized according to their hydrodynamic diameter, polydispersity index (PDI), zeta potential, encapsulation efficiency (EE), loading capacity (LC), and morphology (Fig. 2). All formulations showed a hydrodynamic diameter below 300 nm, and a relatively low PDI (<0.2). The zeta potential of the LNPs was also measured, revealing values below -30 mV. Both the PDI and zeta potential values suggest that the produced formulations have a low tendency to form aggregates^16^. Besides, the encapsulation of CAS does not seem to affect the physical characteristics of the LNPs. The EE and LC of both non-functionalized and functionalized LNPs were measured. CAS-LNPs and F-CAS-LNPs exhibited encapsulation efficiencies of 63% and 83%, respectively. Although functionalized LNPs showed a higher EE, it was not significantly different from the EE of non-functionalized nanoparticles. The LC values were also calculated for both formulations, revealing values between 4% and 6.5%, respectively. The effect of functionalization in the morphology of the CAS-loaded LNPs was also evaluated (Fig. 2a). Both non- and functionalized nanoparticles show a spherical shape and no visible aggregation. These results corroborate the measurements determined by dynamic light scattering.

The physical stability of the formulations under storage conditions was also assessed over 8 weeks (Fig. 2b–e). The hydrodynamic diameter of all formulations remained below 300 nm, which is suitable for a systemic administration route^17^. Interestingly, significant changes in the unloaded LNPs (uLNPs and F-uLNPs) were observed over time, while no changes were observed for CAS-loaded LNPs. Thus, these results suggest that the encapsulation of CAS may contribute to the higher stability of the nanoparticles. During the 8 weeks of study, the PDI and zeta potential showed values below 0.2 and -30 mV, respectively. These low values indicate that the developed LNPs do not tend to form aggregates under storage conditions.

The EE and LC of CAS-loaded LNPs were also measured over time. It is possible to observe that EE decreased to values below 10% for both CAS-LNPs and F-CAS-LNPs, after 8 weeks of study. For the latter, a significant decrease in EE was already observed at week 2. Consequently, the LC values observed at week 8 were significantly lower (<1%) than at week 0. The release of CAS from the nanoparticles is probably due to the amphipathic nature of this lipopeptide, allowing the molecule to diffuse through the lipidic matrix of the developed LNPs^18^.

### In vitro drug release

An in vitro drug release study was conducted to evaluate the release of CAS at a physiological pH (pH = 7.4). The results obtained for the LNPs suspensions (CAS-LNPs and F-CAS-LNPs) and free CAS are shown in Supplementary Fig. 1. The presented data indicates an initial burst release of ~20% after 1 h for both CAS-LNPs and F-CAS-LNPs. After this initial fast release, the LNPs suspensions showed a controlled and sustained release profile, achieving a CAS release of ~70% at 24 h. At 48 h, a significant lower release from F-CAS-LNPs was observed compared to CAS-LNPs and free CAS. Overall, these results suggest that the developed LNPs are suitable carriers of CAS due to the controlled release profile under physiological conditions, preventing the release of drug in the blood circulation, after an intravenous administration.

### In vitro cytocompatibility studies

**Fig. 3:**
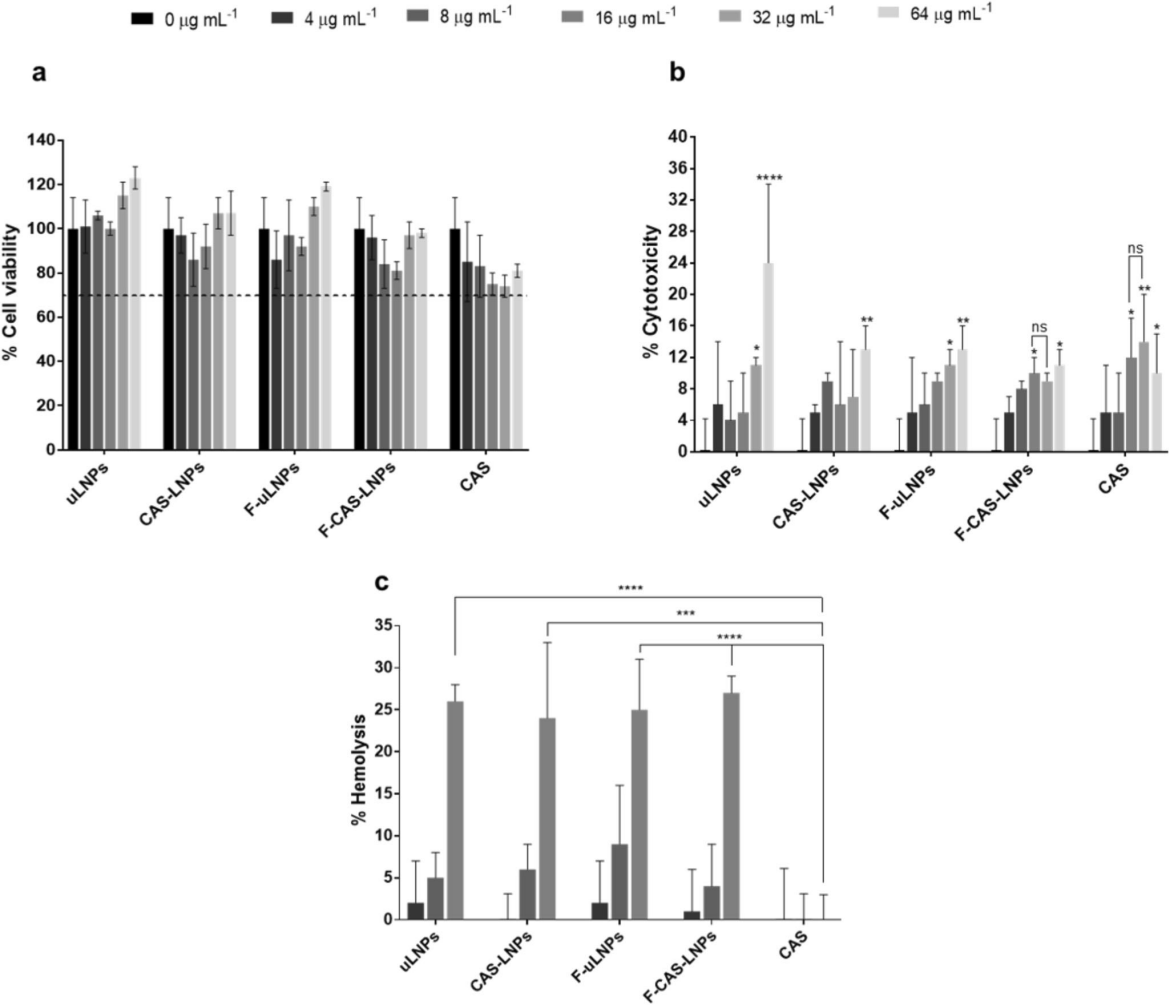
In vitro cytocompatibility studies. **a** MTT and **b** LDH assays using the L929 fibroblast cell line. In these assays, different concentrations of encapsulated and free CAS were assessed (0, 4, 8, 16, 32, and 64 µg mL^−1^). Triton^TM^-100x (1%, v/v) and DMEM were used as negative and positive controls, respectively. **c** Hemolysis assay. Encapsulated and free CAS at the CAS concentrations 4, 8, and 16 µg mL^−1^ were tested. For positive and negative controls, saline solution (0.85%, w/v) and Triton^TM^-100x (1%, v/v) were used, respectively. All values are represented as mean ± SD. *p < 0.05, **p < 0.01, relatively to the positive control. ns, not significant; ***p < 0.001, ****p < 0.0001. Statistical analysis: two-way ANOVA, Tukey’s multiple comparisons test.

Prior to antibacterial and antibiofilm efficacy assays, the cytocompatibility of the developed LNPs was evaluated using a murine fibroblast cell line and human red blood cells (Fig. 3). At all tested concentrations, the LNPs and free CAS showed cell viability above 70% (Fig. 3a), revealing no potential toxicity according to the ISO guidelines^19^. However, the lactate dehydrogenase (LDH) assay (Fig. 3b) showed that at the two highest concentrations tested (32 and 64 µg mL^−1^ of CAS) both unloaded and CAS-loaded formulations exhibited significantly higher cytotoxicity, compared to the control (0 µg mL^−1^). At a concentration of 16 µg mL^−1^, free CAS exhibited cytotoxic effects toward the L929 fibroblast cell line. Hence, encapsulated CAS seems to have lower cytotoxicity than the free compound at the same concentration. This result suggests that not all encapsulated CAS was released from the nanoparticles and, therefore, the fibroblasts were exposed to lower concentrations of CAS over the studied time.

The developed formulations were also evaluated for their potential hemolytic activity (Fig. 3c and Supplementary Fig. 2). At the lowest concentrations tested (4 and 8 µg mL^−1^ of CAS), all formulations showed no significant differences in the hemolysis values, compared to the free compound. In fact, free CAS and the developed LNPs showed a mean hemolysis value below 5%, except for F-uLNPs. At a CAS concentration of 16 µg mL^−1^, it is possible to observe that all nanoformulations exhibited a significantly higher hemolytic activity compared to the free drug, with hemolysis values between 20% and 30%. Despite several literature reports claim that hemolysis values between 5% and 25% are of no concern^20,21^, the ASTM E2524-08 standard states that hemolysis percentages above 5% cause some damage to red blood cells. These results suggest that the hemolytic potential of the formulations is promoted by the vehicle itself rather than the encapsulated drug. The toxicity presented by the vehicle may be due to the use of Tween^®^80 as a surfactant. This nonionic surfactant is widely used in the pharmaceutical industry. Nevertheless, several adverse side effects of Tween^®^80, such as hemolysis, have been reported^22^.

Overall, the developed LNPs suspensions show a safe profile up to a CAS concentration of 8 µg mL^−1^, with no observable toxic effects for either human fibroblasts and red blood cells.

### In vitro antibacterial studies

The effect of the formulations on planktonic *S. aureus* was evaluated by determination of the minimal inhibitory concentration (MIC) and minimal bactericidal concentration (MBC) values for four strains: ATCC 33591 (methicillin-resistant), ATCC 25923 (methicillin-susceptible), ATCC 6538 (methicillin-susceptible), and *Xen36* (methicillin-susceptible). At the tested CAS concentrations (0–64 µg mL^−1^), no inhibition of planktonic bacterial growth was observed after treatment with the formulations, for all strains. For the free CAS, the MIC value was 32 µg mL^−1^ for all tested strains. The MBC value corresponded to the MIC, for the four strains. Although commonly used to treat fungal infections, recent studies report antimicrobial activity of CAS against bacterial strains^7,8^. Siala et al. (2016) reported MIC values between 80 and 160 µg mL^−1^ for several MRSA and MSSA strains^8^. In a more recent study, Sumiyoshi et al. (2020) observed that a CAS concentration of 50 µg mL^−1^ was bactericidal for both MRSA and multidrug-resistant *Pseudomonas aeruginosa*^7^. Thus, the MIC and MBC values obtained for free CAS are supported by previously reported studies. Nevertheless, encapsulated CAS did not show inhibition of bacterial growth at the tested concentrations. This outcome corroborates the hypothesis that not all encapsulated CAS was released from the vehicle at the tested conditions.

### In vitro antibiofilm studies

**Fig. 4:**
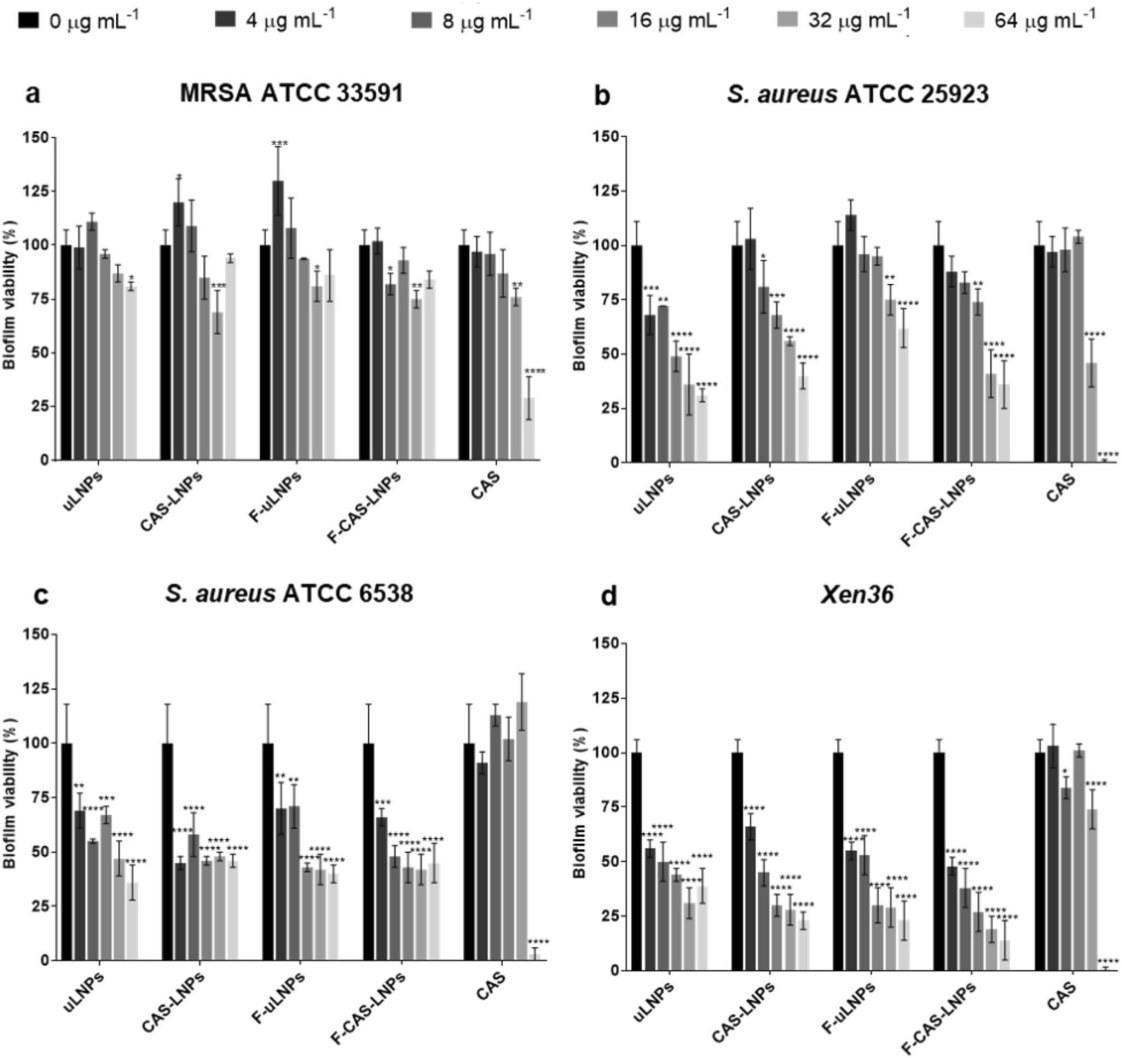
Quantification of biofilm viability by the XTT assay, after a 24 h treatment with LNPs or free CAS at the CAS concentrations of 0, 4, 8, 16, 32, and 64 µg mL^−1^. Prior to the treatment, the biofilms of **a** MRSA ATCC 33591, **b** *S. aureus* ATCC 25923, **c** *S. aureus* ATCC 6538, and **d** the bioluminescent strain *Xen36* were grown in 96-well plates for 24 h. Untreated biofilms (0 µg mL^−1^ of CAS) were used as a positive control. The values are represented as the mean ± SD. *p < 0.05, **p < 0.01, ***p < 0.001, ****p < 0.0001 relatively to 0 µg mL^−1^. Statistical analysis: two-way ANOVA, Dunnett’s multiple comparisons test.

The antibiofilm efficiency of the developed formulations against *S. aureus* strains was evaluated by determining the biofilm viability using the 2,3-Bis(2-methoxy-4-nitro-5-sulfophenyl)-2H-tetrazolium-5-carboxanilide inner salt (XTT) assay, using a microtiter biofilm model (Fig. 4). For this purpose, the LNPs and free CAS were tested at CAS concentrations between 0 and 64 µg mL^−1^. At a cytocompatible concentration (8 µg mL^−1^), only F-CAS-LNPs showed a significant reduction of bacterial viability in MRSA biofilms, compared to the untreated control. However, both unloaded and loaded LNPs showed a higher antibiofilm efficiency against biofilms formed by the remaining three methicillin-susceptible *S. aureus* strains. These results may be a consequence of the increased virulence of MRSA strains, compared to the methicillin-susceptible ones. The presence of resistance genes in MRSA strains promotes their antibiotic resistance, leading to severe infections with high morbidity and mortality rates^23^. Free CAS was also tested against the *S. aureus* biofilms, revealing a significant effect on bacterial viability at the highest concentrations, namely 32 and 64 µg mL^−^^1^ of CAS. Thus, the main effect of the LNPs suspensions on biofilm viability seems to be associated to the composition of the vehicle. This bactericidal effect may be promoted by the presence of Tween^®^80 in the formulations, which was used as a surfactant to produce the LNPs. This compound has antibacterial and antibiofilm properties described against various bacterial species, including *S. aureus* strains^24–26^. In addition, biofilm biomass was also assessed for the *S. aureus* strains in this study (Supplementary Fig. 3). Overall, the crystal violet assay corroborates the results obtained from the XTT assay, with a higher efficacy of the formulations observed for methicillin-susceptible strains.

**Fig. 5:**
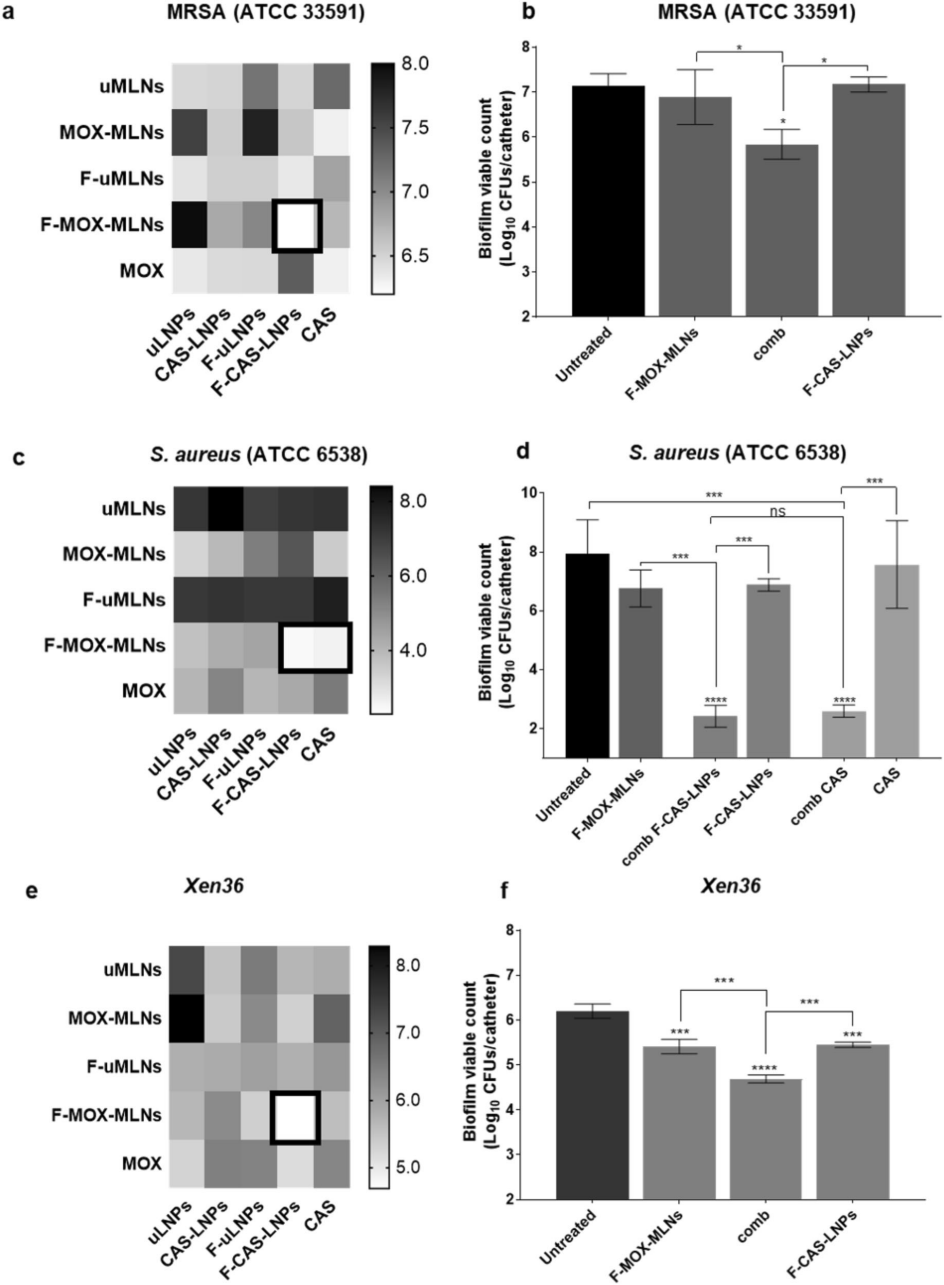
Biofilm viable count of biofilms grown in vitro using the catheter biofilm model. **a**, **b** MRSA ATCC 33591, **c**, **d** *S. aureus* ATCC 6538, and **e**, **f** *Xen36* biofilms grown in vitro in catheters after treatment with the combination of lipid nanosystems. **a**, **c**, and **e** Heat maps represent the effect of the combination of CAS- and MOX-loaded nanosystems on the biofilm viable count (represented as Log_10_ CFUs/catheter). The treatment combinations with lower viable counts are highlighted (black squares). **b**, **f** F-MOX-MLNs and F-CAS-LNPs alone and in combination (comb). **d** F-MOX-MLNs alone and in combination with F-CAS-LNPs (comb F-CAS-LNPs) or CAS (comb CAS). F-CAS-LNPs and CAS alone are also represented. The developed LNPs were assessed at a CAS concentration of 8 µg mL^−1^. MOX-loaded nanosystems were tested at a MOX concentration of 0.5 µg mL^−1^, except for the *S. aureus* ATCC 6538 (1 µg mL^−1^ of MOX). Untreated biofilms were used as a positive control. The values are represented as the mean ± SD for three catheters. *p < 0.05, ***p < 0.001, ****p < 0.0001, relatively to the untreated biofilms. ns, not significant; *p < 0.05, ***p < 0.001. Statistical analysis: one-way ANOVA, Tukey’s multiple comparisons test.

To evaluate the possible combinatory potential of the developed LNPs with antibiotic-loaded nanoparticles, the catheter biofilm model was used. The combination of the two nanosystems was evaluated in three *S. aureus* strains (Fig. 5). For both the MRSA strain and *Xen36*, the combination of the functionalized loaded nanoparticles (F-CAS-LNPs and F-MOX-MLNs) showed the highest potential in reducing the biofilm viability, compared to the untreated control. This combination suggests potential combinatory effects between the two formulations since a significant viability reduction is observed, compared to F-CAS-LNPs and F-MOX-MLNs alone. For the strong *S. aureus* biofilm-forming strain (ATCC 6538), F-MOX-MLNs in combination with F-CAS-LNPs or free CAS showed a very significant reduction of biofilm viability, compared to the untreated control (*p* < 0.0001) and the formulations alone (*p* < 0.001). For this strain, neither F-MOX-MLNs, F-CAS-LNPs, or free CAS alone showed any statistical difference to the untreated control. This outcome suggests that encapsulated and free CAS may contribute to the disruption of the biofilm matrix, exposing a higher number of bacterial cells to the antibiotic. In fact, the potential combinatory effect of CAS and MOX against *S. aureus* biofilms was previously reported^8^. However, in the reported study, higher concentrations of CAS (40 µg mL^−1^) and MOX (10 µg mL^−1^) were required to observe significant biofilm viability reduction compared to the untreated control^8^. The effect of caspofungin against bacterial biofilms is also described in other studies from the literature^7,27^.

Overall, the results showed that a therapeutic approach combining nanosystems loading matrix-disruptive agents, such as caspofungin, and antibiotics, is a step toward the eradication of biofilms.

### In vivo biodistribution study

**Fig. 6:**
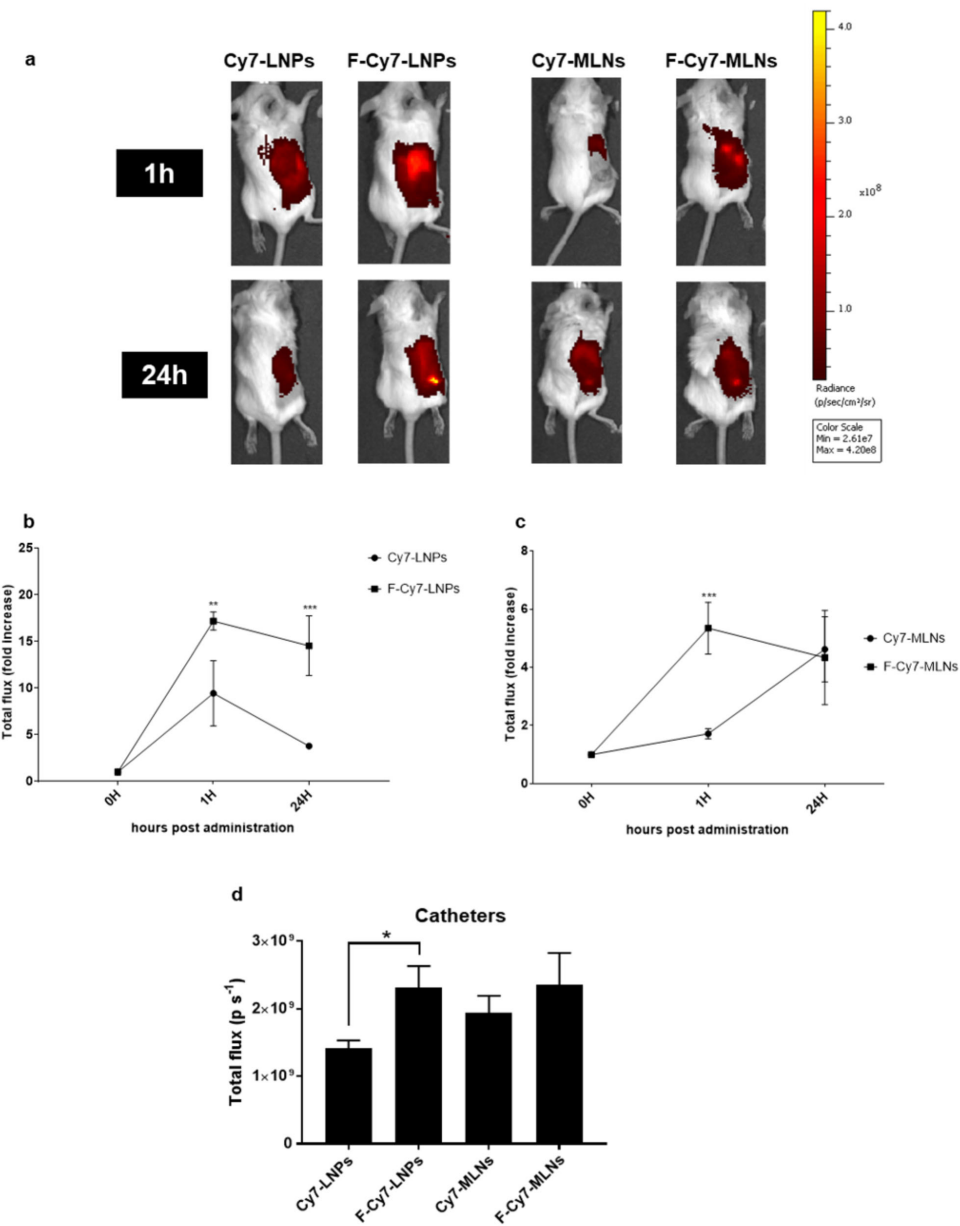
In vivo biodistribution study. **a** Real-time whole-body imaging at different time-points (1 h and 24 h) after intraperitoneal administration of Cy7-loaded LNPs or Cy7-loaded MLNs (non-functionalized or functionalized). Graph of fluorescence signal intensity expressed as *n*-fold increase with respect to 0 h (prior administration), at 1 h and 24 h post-administration of **b** Cy7-loaded LNPs (Cy7-LNPs and F-Cy7-LNPs) and **c** Cy7-loaded MLNs (Cy7-MLNs and F-Cy7-MLNs). The values are represented as the mean ± SD for three animals. **p < 0.001, ***p < 0.001, relatively to 0 h. Statistical analysis: two-way ANOVA, Sidak’s multiple comparisons test. **d** Fluorescence signal intensity of the implanted catheters acquired ex vivo at 24 h post-administration of the Cy7-loaded formulations. Fluorescent signals are expressed in photons per second (p s^−1^). The values are represented as the mean ± SD for three animals (each implanted with four catheters). *p < 0.05. Statistical analysis: two-way ANOVA, Tukey’s multiple comparisons test.

After intraperitoneal administration of both non-functionalized and functionalized LNPs and multiple lipid nanoparticles (MLNs) loaded with cyanine 7 (Cy7), the animals were imaged at two different time points (1 h and 24 h post-administration) to assess the biodistribution. Cy7-loaded LNPs (Cy7-LNPs and F-Cy7-LNPs) have the same lipid composition of CAS-loaded LNPs (CAS-LNPs and F-CAS-LNPs), while Cy7-loaded MLNs (Cy7-MLNs and F-Cy7-MLNs) have the same lipid matrix of MOX-loaded MLNs (MOX-MLNs and F-MOX-MLNs). At 1 h post-administration, it is possible to observe that all formulations accumulated in a specific abdominal region that corresponds to where the infected catheters were previously placed (Fig. 6a). The fast diffusion of the lipid nanosystems across the peritoneal membrane has also been recently reported by Mannucci et al. (2020), which observed a rapid rise of the fluorescence signal in the whole animal body only 30 min after intraperitoneal injection^28^. At 24 h post-administration, a stronger fluorescent signal in the region of the implanted catheters is observable by optical imaging. For the Cy7-loaded LNPs, it is possible to observe significant differences between functionalized and non-functionalized formulations at both 1 h and 24 h post-administration, with a higher accumulation of functionalized nanoparticles in the region surrounding the biofilm (Fig. 6b). Regarding Cy7-loaded MLNs, a significantly higher accumulation of F-Cy7-MLNs compared to Cy7-MLNs is shown in the surroundings of the infected catheters at 1 h post-administration (Fig. 6c). However, this difference is no longer observed after 24 h. The higher accumulation of functionalized nanoparticles is partially due to the PEGylation of these carriers, which avoids recognition and clearance by the host immune system, leading to an increased circulation time in the bloodstream^29,30^. In addition, the use of D-amino acids in the functionalization of the nanoparticles contributes to a higher accumulation in the biofilm region^14^.

The ex vivo acquisition of isolated catheters corroborated the previous results, with a higher accumulation of F-Cy7-LNPs in the catheter pieces than the corresponding non-functionalized formulations (Fig. 6d). Although F-Cy7-MLNs show a higher fluorescent signal than Cy7-MLNs, it is not statistically significant. The accumulation of nanoparticles in the spleen, kidneys, and liver was also evaluated ex vivo (Supplementary Fig. 4). At 24 h post-administration, all formulations were mainly accumulated in the kidneys and liver, while a considerably lower signal was observed in the spleen. These findings revealed that after 24 h, a high number of nanoparticles was cleared by the reticuloendothelial system (RES), which led to accumulation in the liver^30^. The high intensity of the fluorescence signal in the kidneys also shows that these nanocarriers can be eliminated by the renal clearance pathway, which substantially reduces the potential toxicity of the nanoparticles, compared with the RES clearance. Thus, the high renal clearance observed for the developed LNPs and MLNs may suggest that these nanosystems present a good biocompatibility profile^31^.

### In vivo antibiofilm efficacy study

**Fig. 7:**
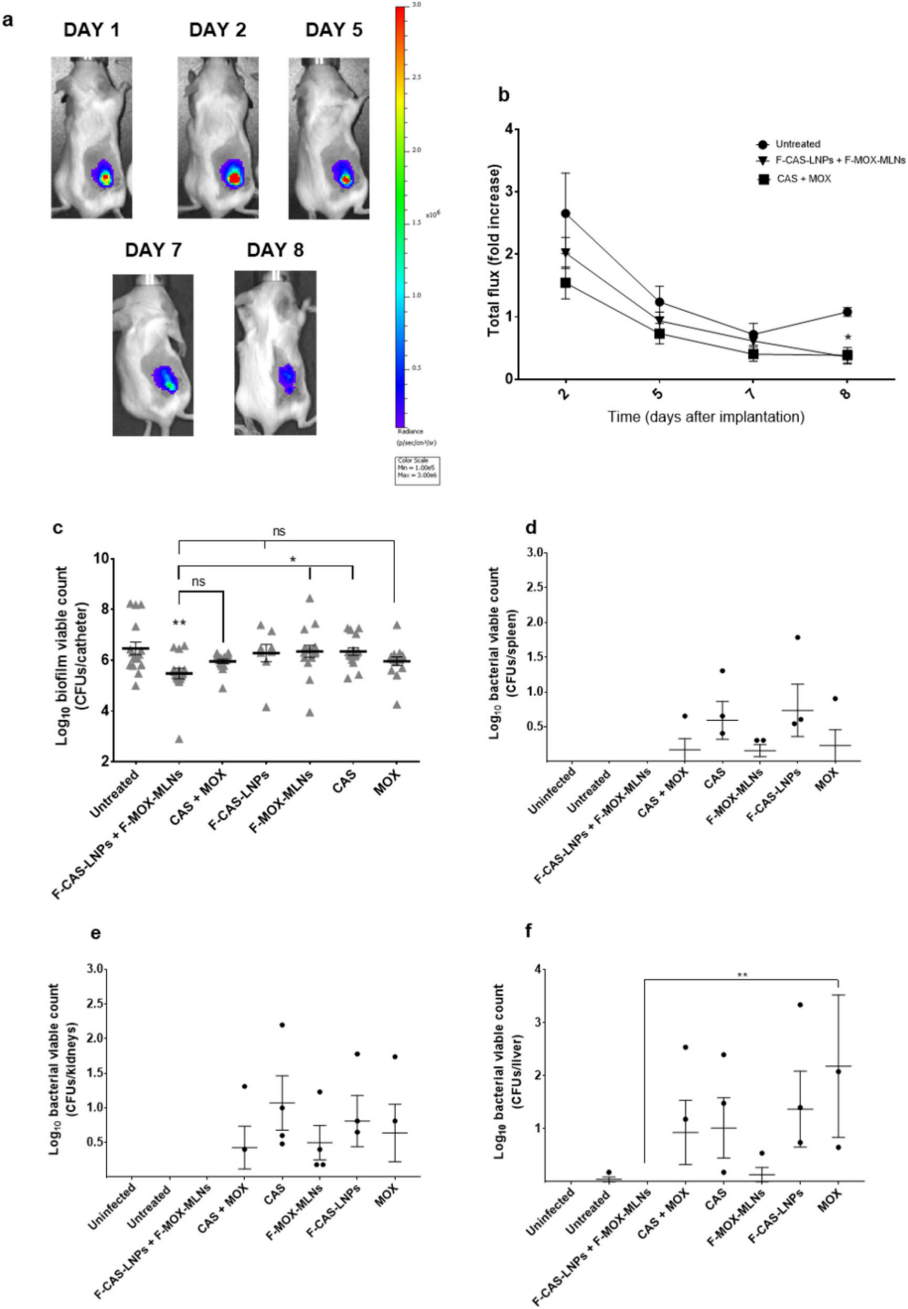
In vivo antibiofilm activity of the combination of CAS-loaded and MOX-loaded nanosystems against the bioluminescent strain *Xen36*. Infected catheters were implanted at day 0 in the back of mice treated 24 h after implantation and for 7 days. **a** Representative bioluminescence images at different time points (1, 2, 5, 7, and 8 days after implantation) of the group treated with the combination of F-CAS-LNPs with F-MOX-MLNs. On day 1, mice were imaged before the treatment. **b** Graph of bioluminescence signal intensity expressed as *n*-fold increase with respect to day 1 after implantation (prior to treatment), at different time points (2, 5, 7, and 8 days after implantation). The values are represented as the mean ± SEM for four animals. *p < 0.05 relatively to the untreated control at the same time point. Statistical analysis: one-way ANOVA, Dunnett’s multiple comparisons test. **c** Biofilm viable count retrieved from implanted catheters after 7 days of treatment. The catheters were recovered after sacrifice on day 8 after implantation. The values are represented as the mean ± SEM for four animals (each implanted with four catheters). **p < 0.01, relatively to the untreated control. ns, not significant; *p < 0.05. Statistical analysis: one-way ANOVA, Tukey’s multiple comparisons test. Bacterial viable count in **d** spleen, **e** kidneys, and **f** liver of mice. The organs were collected on day 8 after implantation. **p < 0.01. Statistical analysis: two-way ANOVA, Tukey’s multiple comparisons test.

To evaluate the in vivo efficacy of CAS-loaded and MOX-loaded nanosystems alone or in combination, mice were implanted with *Xen36*-infected catheters and further treated for 7 days. The biofilms formed in vivo were monitored using bioluminescence imaging from day 1 (prior to treatment) to day 8 after implantation (Fig. 7a, b and Supplementary Fig. 5). Untreated mice showed a linear signal intensity from day 5 to day 8, while mice treated with the combination of F-CAS-LNPs with F-MOX-MLNs, or the combination of the free compounds exhibited a decrease in signal intensity over time. On day 8 post-implantation, a significantly lower bioluminescence intensity signal was observed for both treatments, compared with the untreated control. At this time point, implanted catheters were recovered from sacrificed mice to quantify biofilm viability (Fig. 7c). Only the combination of both nanosystems (F-CAS-LNPs and F-MOX-MLNs) revealed a significant decrease in the viable count compared to the untreated control (difference of 1-log_10_). However, the treatment with both nanosystems combined did not show a significant difference compared to the treatment with the combination of the free compounds (CAS + MOX).

The potential dissemination of bacterial cells into vital organs after treatment was also assessed by quantifying the bacterial viability in the spleen, kidneys, and liver (Fig. 7d–f). Interestingly, only the mice treated with F-CAS-LNPs in combination with F-MOX-MLNs did not show dissemination in these organs. The nanosystems alone and the free compounds alone or in combination (CAS + MOX) promoted the dissemination of bacterial cells into the three organs, with a higher bacterial load observed in the liver. The dissemination of bacteria to these organs is highly concerning since it may contribute to the survival of the pathogen. When *S. aureus* cells reach the liver, they are rapidly phagocyted by Kupffer cells. However, it is reported that part of the bacterial cells may survive and proliferate intracellularly, remaining undetected by neutrophils^32,33^. Additionally, intracellular *S. aureus* is protected from antibiotic activity since most antibiotics do not reach the intraphagocyte niche^32^. Eventually, Kupffer cells lyse, leading to the release of bacteria into the circulation. This process promotes the dissemination of *S. aureus* to other organs, such as kidneys, where they can form abscesses^32,33^.

Overall, these results suggest that the combination of F-CAS-LNPs with F-MOX-MLNs is a potentially safe antibiofilm therapeutic approach, which does not promote the dissemination of bacterial cells from the biofilm into vital organs.

## Discussion

The CAS-loaded LNPs developed in this work presented a spherical morphology, with a mean hydrodynamic diameter below 300 nm, a low PDI (<0.2), and a highly negative zeta potential, which reveals a low tendency to form aggregates. The EE and LC were satisfactorily high during 2 weeks of storage, with values above 60% and 4%, respectively. At 8 weeks under storage conditions (4 °C), most of the encapsulated CAS was released from the nanoparticles by diffusion. These physical characteristics of the developed formulations are suitable for an intravenous administration route and to reach and penetrate the biofilm matrix, since several studies suggest that nanosystems with low hydrodynamic diameters are able to reach deeper regions of the biofilm^34,35^. Additionally, the developed LNPs suspensions presented adequate release properties for intravenous administration, with an initial burst release followed by a sustained and controlled release profile, at a physiological pH.

The cytocompatibility of the developed LNPs was evaluated against fibroblasts (L929 cell line) and human red blood cells. Cell viability studies revealed that the formulations did not exhibit cytotoxic effects against fibroblasts up to a CAS concentration of 16 µg mL^−1^. However, the formulations showed a significantly higher hemolytic activity at this concentration than the free compound. The higher hemolytic activity of the formulations may be due to the presence of Tween^®^80, since it has been reported that this surfactant promotes hemolysis^22^. Hence, the developed LNPs showed a safe profile up to a CAS concentration of 8 µg mL^−1^.

The in vitro antibiofilm activity of the CAS-loaded LNPs was firstly evaluated using the microtiter plate biofilm model. At a cytocompatible concentration (8 µg mL^−1^ of CAS), only the functionalized CAS-loaded formulation (F-CAS-LNPs) showed a significant biofilm viability reduction against MRSA. However, for the methicillin-susceptible strains, all formulations significantly reduced the viability at the same concentration. It is hypothesized that this difference between the strains is mainly due to the increased virulence of the MRSA strain, compared to the remaining studied strains^23^, leading to lower efficiency of the LNPs. The biofilm viability after treatment with CAS-loaded LNPs alone or in combination with MOX-loaded MLNs was further evaluated in an in vitro catheter biofilm model. Interestingly, for the three strains studied, the combination of functionalized loaded nanoparticles (F-CAS-LNPs and F-MOX-MLNs) showed the highest reduction in the biofilm viable count. This potential combinatory effect between CAS and MOX is supported by a previous report in the literature^8^. In the work of Siala et al. (2016), higher concentrations of CAS (40 µg mL^−1^) and MOX (10 µg mL^−1^) were required to observe significant biofilm viability reduction compared to the untreated control^8^. The potential combinatory effect between these two nanosystems was further confirmed by in vivo efficacy studies. The combination of F-CAS-LNPs with F-MOX-MLNs showed a decrease in the biofilm viable count of implanted catheters recovered from mice, compared to the untreated control. Besides, the treatment with this combination showed no signs of dissemination of bacterial cells into vital organs, such as the spleen, kidneys, and liver. Additionally, in vivo biodistribution studies showed that the two nanosystems reached the targeted site, accumulating in the biofilm region, even 24 h post-administration. The higher accumulation of functionalized LNPs and MLNs (F-Cy7-LNPs and F-Cy7-MLNs) is likely due to the use of PEG, which avoids recognition and clearance by the host immune system^29,30^, and D-amino acids, which is reported to target the biofilm matrix^14^. Besides, a high renal clearance observed for the developed nanosystems suggests a good biocompatibility profile^31^. The combination of matrix-disruptive agents with antibiotics to fight bacterial biofilms has been recently highlighted in the literature as an alternative antibiofilm therapy to the administration of conventional antibiotics at high doses for long periods. In addition, the use of nanocarriers may play a critical role to target encapsulated drugs to the biofilm site and increase penetration within the biofilm matrix, leading to the release of encapsulated contents closer to the bacterial cells. Besides, other technologies such as photodynamic therapy and ultrasounds are currently being applied as tools in combination with nanoparticles, to further improve current antibiofilm therapies^4^. Siala et al. (2016) explored the combination of free CAS and MOX to eradicate *S. aureus* biofilms; however, a concern of the authors was the potential effect of the administration of free CAS in the fungal flora^8^. This limitation is overcome by the encapsulation and targeting of CAS into nanoparticles to the region of the bacterial biofilm. In a previous study, we also observed promising results in combining MOX-loaded nanoparticles with nanoparticles loaded with *N*-acetyl cysteine (matrix-disruptive agent)^36^. Overall, this work presents a novel non-disseminative nano-strategy combining functionalized nanosystems encapsulating matrix-disruptive (CAS) and bactericidal (MOX) agents, as a tool to improve the therapy against *S. aureus* biofilms.

## Methods

### Materials

The lipid cetyl palmitate was a kind gift from Gattefossé (Gattefossé SAS, Saint-Priest, France). DSPE-PEG2000-NH_2_ (1,2-distearoyl-*sn*-glycero-3-phosphoethanolamine-N-[amino(polyethylene glycol)-2000] (ammonium salt)) was purchased from Avanti Polar Lipids Inc. (Alabaster, Alabama, USA). D-Phenylalanine, D-Proline, D-Tyrosine, triethylamine (TEA), dicyclohexylcarbodiimide (DCC), N-hydroxysuccinimide (NHS), sodium hydroxide (NaOH), sodium chloride, moxifloxacin hydrochloride (MOX), farnesol 95%, Tween^®^80, trifluoroacetic acid (TFA), Triton^TM^-100x, thiazolyl blue tetrazolium bromide (MTT), D-(+)-glucose monohydrate >99%, Müller-Hinton broth cation adjusted (MHB-Ca^2+^), 2,3-Bis(2-methoxy-4-nitro-5-sulfophenyl)-2H-tetrazolium-5-carboxanilide inner salt (XTT), menadione crystalline, and crystal violet dye were obtained from Sigma-Aldrich (St. Louis, Missouri, USA). Miglyol^®^812 and Span^®^80 were purchased from Acofarma (Madrid, Spain). Chloroform, dimethyl sulfoxide (DMSO), methanol, acetonitrile, and acetic acid glacial were obtained from VWR International LLC (Radnor, Pennsylvania, USA). Dulbecco’s Modified Eagle’s Medium (DMEM), trypsin-EDTA (1×), Penicillin-Streptomycin (PenStrep), Dulbecco’s Phosphate Buffered Saline 10x pH 7.4 (PBS), and Fetal Bovine Serum (FBS) were purchased from Gibco by Life Technologies (Paisley, UK). Lactate dehydrogenase (LDH) Cytotoxicity Detection Kit was obtained from Takara Bio Inc. (Shiga, Japan). L929 cells (passage number 19–20) were acquired from the European Collection of Authenticated Cell Cultures (ECACC, Salisbury, UK). BD Tryptic soy broth (TSB) and Difco Granulated Agar were acquired from Becton Dickinson (Franklin Lakes, New Jersey, USA). Cyanine 7 (Cy7) was purchased from Lumiprobe GmbH (Hannover, Germany). The isoflurane (Iso-Vet 1000 mg/g) was acquired from Dechra Veterinary Products NV (Lille, Belgium). All components were used without further purification.

The *S. aureus* strains ATCC 33591 (methicillin-resistant), ATCC 25923 (methicillin-susceptible), and ATCC 6538 (methicillin-susceptible) were acquired from ATCC (Manassas, Virginia, USA). The bioluminescent strain *Xen36* (Caliper Life Sciences, Waltham, Massachusetts, USA) was derived from the *S. aureus* ATCC 49525. This strain expresses a stable copy of a modified *Photorhabdus luminescens luxABCDE* operon^37,38^.

### Preparation of the CAS-loaded LNPs

The formulations were produced by the double emulsion method. In this method, CAS (5 mg) was dissolved in an aqueous solution of NaOH (400 µL of NaOH 1 M plus 600 µL of type I water). For the unloaded LNPs, CAS was not added to the aqueous solution. The aqueous solution was then added to the solid lipid cetyl palmitate (65 mg), previously dissolved in 2 mL of chloroform. The mixture was sonicated for 30 s at an amplitude of 70%, using a VCX-130 Vibra-Cell sonicator (Sonics & Materials Inc, Newtown, Connecticut, USA) with a CV-18 probe (130 W, 20 kHz). For the functionalized formulations, 1 mg of each D-amino acids conjugate (D-Phenylalanine, D-Proline, and D-Tyrosine) were dissolved in 100 µL of chloroform and added to the primary emulsion. The synthesis of these conjugates is described below. Then, a Tween^®^80 (25 mg mL^−1^, 4 mL) solution was added, and the emulsion was sonicated for 3 min at an amplitude of 80%. Finally, a Tween^®^80 (13.75 mg mL^−1^, 4 mL) solution was added, and the final LNPs suspension was placed in a stirring plate at room temperature, under agitation, to evaporate the remaining chloroform.

The surface of the lipid nanoparticles was functionalized with DSPE-PEG_2000_-NH_2_-D-amino acid (D-Phenylalanine, D-Proline, and D-Tyrosine) conjugates^39^. To activate the D-amino acids, 62.5 mg of each D-amino acid was added to a mixture of 5 mL of DMSO and 62.5 μL of TEA and stirred in the dark, overnight, in anhydrous conditions. Then, 62.5 mg of DCC and 65 mg of NHS were added to the previous mixture and stirred in the dark, overnight. The removal of the side product dicyclohexylurea was performed using a 0.45 μm filter (Ministart, pore size 450 nm, cellulose acetate membrane, Sartorius Stedim Biotech, Germany). Afterward, DMSO and TEA were left to evaporate in vacuum conditions. To each activated D-amino acid solution (2 mL), 1 mL of DMSO and 50 mg of DSPE-PEG_2000_-NH_2_ were added and stirred in the dark, overnight, in anhydrous conditions. Afterward, the evaporation of DMSO was performed in vacuum conditions, and 6 mL of type I water were added. The conjugates were purified using the dialysis diffusion technique (Spectra/Por3 Dialysis Membrane, 3.5 kD, Spectrum Laboratories, Inc., California, USA). In this technique, 1 L of type I water was used for 48 h to remove the unconjugated D-amino acids. The conjugates were lyophilized (LyoQuest −85 plus v.407, Telstar, Barcelona, Spain) and stored at −20 °C^39^.

### Physical characterization of LNPs

The hydrodynamic diameter and PDI were measured using a Particle Size Analyzer (Brookhaven Instruments Corporation, Software: Particle Sizing v.5 Brookhaven Instruments, Holtsville, New York, USA). The zeta potential of the developed LNPs was determined using the Zeta Potential Analyzer (ZetaPALS, Brookhaven Instruments Corporation, Software: PALS Zeta Potential Analyzer v.5 Brookhaven Instruments, Holtsville, New York, USA). The system operated with an incidence light angle of 90˚, at room temperature. Prior to the measurements, the LNPs were diluted (1:100) in type I water.

The morphology of the produced LNPs was assessed by TEM. For this purpose, the formulations were diluted (1:100) in type I water. Then, 10 µL of the diluted LNPs were placed onto a copper-mesh grid for 2 min. The excess of LNPs was then removed, and 10 µL of 0.75% (w/v) uranyl acetate solution were added to the grid for 30 s (negative staining). The samples were observed in a JEM-1400 transmission electron microscope (JEOL Ltd., Tokyo, Japan) with an acceleration voltage of 80 kV.

### Determination of the encapsulation efficiency and loading capacity

The EE was determined using the reverse-phase high-performance liquid chromatography (HPLC), by direct measurement of the entrapped CAS. The diluted LNPs (80 µL of LNPs in 1920 µL of type I water) were transferred to an Amicon^®^Ultra-4 Centrifugal Filter Device 50 kDa (MERCK Millipore, Ltd, Dublin, Ireland) and centrifuged at 524 *g* (Allegra X-15R Centrifuge, Beckman Coulter, Brea, California, USA) until complete separation of the LNPs from the supernatant. The pellet was collected and added to 500 µL of acetonitrile, to dissolve the LNPs. The lipids were separated from the dissolved CAS by centrifugation at 30 000 *g* for 30 min. Then, 350 µL of supernatant containing dissolved CAS was added to 650 µL of buffer, composed of an aqueous solution of 0.1% of TFA (v/v), adjusted to pH 3 with TEA.

The chromatographic conditions were adapted from the literature^40^. The HPLC system (Jasco, Tokyo, Japan) was composed of one high-pressure pump (PU-4180), a refrigerated automated injector (AS-4050), and a fluorescence detector (FP-4025) set at 220 nm and 304 nm for excitation and emission, respectively. A sample volume of 20 μL was injected. The chromatographic separation was obtained using a Gemini NX C18 110 Å (150 × 4.60 mm, 5 μm) column from Phenomenex (Torrance, California, USA). The isocratic mobile phase consisted of a 65:35 (v/v) mixture of the buffer mentioned above and acetonitrile. At a flow rate of 0.9 mL min^−1^, the retention time of CAS was 9.5 min. A calibration curve of CAS (0.002 – 0.04 mg mL^−1^) was used for quantification (Supplementary Fig. 6).

The EE was calculated as follows:

1 $$EE\,\left( {{{\mathrm{\% }}}} \right) = \frac{{loaded\,CAS\,amount}}{{total\,CAS\,amount}} \times 100$$

The LC was calculated using the EE, as follows:

2 $$LC\,\left( {{{\mathrm{\% }}}} \right) = \frac{{EE \times total\,CAS\,amount}}{{total\,solid\,lipid\,amount}} \times 100$$

### Evaluation of the storage stability of the LNPs suspensions

The LNPs formulations were stored at 4 °C for 8 weeks. During this time, the physicochemical storage stability was assessed by measuring hydrodynamic diameter, PDI, zeta potential, EE, and LC. The measurements were performed using the methods described in the previous sections.

### In vitro drug release

In vitro drug release in physiological pH (PBS, pH 7.4) to simulate the blood circulation and normal tissues was assessed using the dialysis diffusion method under sink conditions. At 37 °C, under agitation, 1 mL of LNPs or free CAS were added to a cellulose dialysis bag (Spectra/Por3 Dialysis Membrane, 6 − 8 kD, Spectrum Laboratories, Inc., Rancho Dominguez, California, USA). PBS (60 mL) was used as dissolution medium. At defined timepoints (0.5, 1, 2, 3, 5, 8, 24, and 48 h), aliquots of 80 µL LNPs or free CAS were collected from the the dialysis bag. The amount of CAS released was then quantified using the reverse-phase HPLC, by direct measurement, as previously described in “Determination of the encapsulation efficiency and loading capacity”.

### In vitro cytocompatibility studies

The effect of the developed LNPs on cell viability was evaluated in the L929 cell line (murine fibroblasts), as recommended by the ISO international standard 10993–5:2009 for cytocompatibility assessment studies^41^. Briefly, the cells were cultured in DMEM supplemented with 10% (v/v) FBS and 1% (v/v) PenStrep at 37 °C, 5% CO_2_ atmosphere. Briefly, after 80 – 90% confluence, cells were seeded in 96-well plates at a density of 5 ×10^4^ cells/well and let to grow overnight at 37 °C, 5% CO_2_ atmosphere. Afterward, the cells were treated with LNPs or free CAS at different CAS concentrations (0, 4, 8, 16, 32, and 64 µg mL^−1^) for 24 h, at 37 °C, 5% CO_2_. Cells incubated with DMEM and Triton^TM^-100x (2%, v/v) were used as positive and negative controls, respectively.

After the treatment, the medium in each well was replaced by the MTT solution (0.5 mg mL^−1^, 100 µl) and incubated for 2 h, at 37 °C, 5% CO_2_. Then, the MTT solution was removed, and the formazan crystals were dissolved in 100 µL of DMSO. The medium collected after the treatment was further centrifuged at 250 *g* for 10 min (Centrifuge 5810 R, Eppendorf, Hamburg, Germany). The supernatant was collected to quantify the LDH using the LDH cytotoxicity kit, according to the instructions from the manufacturer. For both MTT and LDH assays, the absorbance was measured at 490 and 690 nm using a microplate reader (BioTek Instruments Inc., Synergy HT, Software: Gen5 v1.08.4, BioTek Instruments Inc, Winooski, Vermont, USA). The latter wavelength was used to remove the background interference in the measurements.

Additionally, human red blood cells, kindly donated by Serviço de Hematologia do Centro Hospitalar do Porto, were used to assess the hemolytic activity of the developed LNPs. This procedure was in accordance with the principles of the Declaration of Helsinki. The protocol was adapted from the literature^42^. Briefly, blood was centrifuged at 955 *g* for 5 min, at 4 °C (Allegra X-15R Centrifuge, Beckman Coulter, Brea, California, USA), to separate the red blood cells from the remaining components of the blood. Afterward, red blood cells were washed three times with saline solution 0.85% (w/v) and diluted to a volume fraction of 4%. Diluted red blood cells (100 µL) were incubated with 100 µL of LNPs or free CAS for 1 h, at 37 °C. The LNPs and free CAS were tested at the same CAS concentrations used for the MTT and LDH assays described above. Afterward, the supernatant from each well was collected, and the absorbance was read at 540 nm and 690 nm, using a microplate reader (BioTek Instruments Inc., Cytation, Software: Gen5 v1.08.4, BioTek Instruments Inc., Winooski, Vermont, USA). The percentage of hemolysis was calculated according to the following formula:

3 $$Hemolysis\left( {{{\mathrm{\% }}}} \right) = \frac{{Abs\,(sample) - Abs\,( - )}}{{Abs\,\left( + \right) - Abs\,( - )}} \times 100$$

where Abs (sample), Abs (−) and Abs (+) are the absorbances of the sample, the negative control (saline solution 0.85%, w/v) and the positive control (Triton^TM^-100x, 1% v/v), respectively.

### In vitro antibacterial studies

The effect of the developed LNPs on planktonic bacteria was assessed using the well-known micro-broth dilution assay^43^. This method follows CLSI^44^ and EUCAST^45^ guidelines. In brief, an overnight bacterial culture was diluted to a concentration of 2.0 × 10^5^ CFUs mL^–1^ in MHB-Ca^2+^ medium. Then, the bacterial suspension was treated with LNPs or free CAS for 24 h, at 37 °C, in a 96-well round-bottom microtiter plate (Greiner Bio One, Kremsmünster, Austria). The developed LNPs and free CAS were tested at the same concentrations used for the cytocompatibility studies (in “In vitro cytocompatibility studies”). The MIC was recorded as the lowest concentration, which inhibited the visual growth of bacteria, while MBC was defined as the lowest concentration resulting in the death of 99.9% of the initial inoculum. The MBC was further determined by spot-plate (10 µL) each well with no visual bacterial growth on TSA plates. The TSA plates were incubated at 37 °C, overnight, followed by CFUs counting.

### Microtiter plate biofilm model

Biofilm formation and growth conditions were adapted from the literature^8,46^. Overnight bacterial cultures were adjusted to an optical density of 600 nm (OD_600nm_) of 0.1 in TSB 0.6x supplemented with 0.2% (w/v) glucose. Then, 200 µL of the adjusted inoculum were added to each well from a 96-well flat-bottom microtiter plate (Greiner Bio One, Kremsmünster, Austria) and incubated for 90 min, at 37 °C. Afterward, the medium was removed, and the wells were washed twice with PBS 1x, to remove non-adhered cells. Subsequently, 200 µL of fresh medium were added to each well. The biofilms were left to grow for 24 h, at 37 °C. Prior to treatment, the 24h-old biofilms were washed twice with PBS 1x. The developed LNPs and free CAS were tested at several CAS concentrations (0, 4, 8, 16, 32, and 64 µg mL^−1^). This procedure was used for the following assays.

#### Biofilm metabolic activity assessment

The metabolic activity of the bacterial cells within the biofilm was assessed through the XTT assay^47^. After the treatment, the wells were washed once with PBS 1x, and 100 µL of XTT solution (1 mg mL^−1^, supplemented with 1 µM menadione) were added. Then, the 96-well plates were incubated in the dark, at 37 °C for 30 min, with shaking (250 rpm) (VWR Incubating Microplate Shaker, VWR International LLC, Radnor, Pennsylvania, USA). The absorbance was read at 490 nm using a microplate reader (BioTek Instruments Inc., Synergy HT, Software: Gen5 v1.08.4, BioTek Instruments Inc., Winooski, Vermont, USA).

#### Biofilm biomass study

Biofilm biomass quantification was assessed through the crystal violet assay based on the reported protocol^48^. After the treatment with the LNPs, the biofilms were washed once with PBS 1x and fixed with 200 µL of 99% methanol for 15 min. Then, the methanol was removed from the wells, and the biofilms were left to dry until complete evaporation of the organic solvent. To stain the biofilms, 200 µL of crystal violet solution (1%, w/v) were added to the wells for 5 min, at room temperature. The excess of dye was further removed by washing the stained biofilms with type I water. Finally, 200 µL of glacial acetic acid 33% (v/v) were added to the wells. The plates were incubated at room temperature for 15 min, with shaking (250 rpm), in an incubating microplate shaker (VWR International LLC, Radnor, Pennsylvania, USA). The absorbance was read at 570 nm using a microplate reader (BioTek Instruments Inc., Synergy HT, Software: Gen5 v1.08.4, BioTek Instruments Inc., Winooski, Vermont, USA).

### Catheter biofilm model

The potential antibiofilm effect of the developed LNPs in combination with an antibiotic-loaded nanosystem was further evaluated in the catheter biofilm model. For this purpose, MOX-loaded MLNs were produced^36^. The lipid phase of the MLNs, constituted by the solid lipid cetyl palmitate (135 mg), the lipid liquid Miglyol^®^812 (305 mg), the lipophilic surfactant Span^®^80 (115 mg), and farnesol (6 mg), was heated at a temperature above the melting point of the solid lipid (60˚C) in a water bath. At this temperature, 5 mg of MOX were dissolved in 650 µL of type I water. The unloaded MLNs were prepared by replacing the drug solution with type I water. Afterward, the aqueous solution was added to the melted lipid phase and sonicated at 70% amplitude for 3 min, using a VCX-130 Vibra-Cell^TM^ sonicator (Sonics & Materials Inc, Newtown, Connecticut, USA), with a CV-18 probe (130 W, 20 kHz). After this primary emulsion cooled down at room temperature, the hydrophilic surfactant Tween^®^80 (80 mg) was added. D-amino acid conjugates (D-Phenylalanine, D-Proline, and D-Tyrosine) were also added in the functionalized formulations (1 mg each conjugate). The synthesis of these conjugates is described in more detail in “ Preparation of the CAS-loaded LNPs”. The emulsion was then heated for 5 min at 60˚C and sonicated at 80% amplitude for 3 min^36^.

The biofilms were grown using an optimized in vitro catheter biofilm model was previously reported^8^. First, triple-lumen polyurethane central venous catheters (Certofix duo/trio; B. Braun Melsungen AG, Melsungen, Germany) were cut into 1 cm long pieces. The pieces were incubated overnight, at 37 °C, with 100% fetal bovine serum. The bacterial overnight culture was adjusted to an OD_600 nm_ of 0.1 in TSB 0.6x supplemented with 0.2% glucose (w/v). Then, each catheter piece was incubated with 1 mL of the adjusted bacterial inoculum, for 90 min, at 37 °C, in a 24-well plate for suspension cultures (Greiner Bio One, Kremsmünster, Austria). After the adhesion step, non-adherent bacteria were removed by two washing steps with 1 mL of PBS 1x. The washed catheters were submerged in 1 mL of fresh medium and incubated for 24 h, at 37 °C, to allow the formation of mature biofilms. Afterward, biofilms were treated for 24 h, at 37 °C. For this purpose, the developed LNPs or free CAS were tested at a CAS concentration of 8 µg mL^−1^. MOX-loaded MLNs and respective unloaded formulations were tested at a MOX concentration of 0.5 µg mL^−1^ (for MRSA ATCC 33591 and *Xen36* strains) and 1.0 µg mL^−1^ (for the strong biofilm-forming ATCC 6538 strain). Free MOX was tested in the same conditions. After the treatment, catheter pieces were washed twice with PBS 1x and sonicated (Branson 5510 Ultrasonics bath, Marshall Scientific, Hampton, New Hampshire, USA) at room temperature for 10 min in 200 µL of PBS 1x, to disperse the biofilms. The samples were serially diluted in PBS 1x and spot-plated (10 µL) on TSA plates. The plates were incubated overnight at 37 °C to allow CFUs counting.

### Murine subcutaneous biofilm model

Animal experiments were performed under the KU Leuven animal care guidelines and were approved by the KU Leuven Ethics Committee (project number P019/2021). The murine subcutaneous biofilm model used for the in vivo studies is detailed in the literature^8,49^. Female pathogen-free 8-week-old BALB/c mice (Janvier Labs, Le Genest-Saint-Isle, France) were kept in ventilated cages and provided with food and water *ad libitum*. Briefly, serum-coated catheter pieces (1 cm long) were incubated with the bioluminescent strain *Xen36* for 90 min at 37 °C, to allow adhesion of bacterial cells, as described in “Catheter biofilm model”. After this incubation time, the catheter pieces were washed twice with PBS 1x and subcutaneously implanted in the back of the mice. For this purpose, the mice were anesthetized with a mixture of isoflurane in oxygen (1.5–2%, v/v), and their lower back was shaved and disinfected with ethanol 70%. A small incision in the right side was performed to create a subcutaneous pocket where 4 catheter pieces were placed per mouse. The incision was closed using surgical glue (3 M Vetbond Tissue Adhesive, 3 M, St. Paul, Minnesota, USA), and the biofilms were allowed to grow into mature structures for 24 h, prior to treatment.

### Biodistribution study

For the biodistribution study, LNPs loaded with Cy7 were prepared according to the protocol described for unloaded LNPs in “Preparation of the CAS-loaded LNPs”. For this purpose, Cy7 at a concentration of 0.2% (w/w) of the solid lipid and surfactant was dissolved in chloroform and added to the lipid phase. Both non-functionalized and functionalized Cy7-loaded LNPs were produced.

Cy7-loaded MLNs were prepared based on the protocol described in “Catheter biofilm model”. Briefly, the solid lipid cetyl palmitate (135 mg), the surfactant Span^®^80 (115 mg), and farnesol (6 mg) were heated at 60 °C in a water bath. Cy7 at a concentration of 0.1% (w/w) of the solid lipid and surfactants was added to the liquid lipid Miglyol^®^812 (305 mg) and pre-heated. Afterward, the dye solubilized in the liquid lipid was added to the melted lipid phase. Simultaneously, 650 µL of type I water was added and the mixture was sonicated for 3 min at 70%. The primary emulsion was left to cool down at room temperature. Then, 80 mg of Tween^®^80 were added. For the functionalized formulation, 1 mg of each D-amino acid conjugate (D-Phenylalanine, D-Proline, and D-Tyrosine) were added. The emulsion was then heated for 5 min at 60 °C and sonicated at 80% amplitude for 3 min.

To assess in vivo biodistribution, the non-functionalized and functionalized formulations were administered intraperitoneally to infected mice (*n* = *3* per group). The volume of the intraperitoneal injection per mouse was 250 µL. Cy7-loaded LNPs and Cy7-loaded MLNs were administered at a solid lipid concentration of 52 mg kg^−1^ and 135 mg kg^−1^ of body weight, respectively. The animals were imaged prior, 1 h, and 24 h post-administration of the formulations. For this purpose, mice were anesthetized using a gas mixture of isoflurane in oxygen (1.5 to 2%, v/v) and placed in the In Vivo Imaging System (IVIS Spectrum, Perkin-Elmer, Waltham, Massachusetts, USA). Frames were acquired with the fields of view of 23 cm. Bioluminescence imaging was acquired using 40 s exposure time, binning 2, f/stop = 1. Fluorescence images (l_exc_: 760 nm, l_em_: 780 nm) were acquired using an exposure time of 0.3 s, binning 2, f/stop = 2. After 24 h, the animals were anesthetized with a mixture of isoflurane in oxygen (1.5–2%, v/v) and euthanized by cervical dislocation, and the catheters and organs (liver, spleen, and kidneys) were imaged. Frames with fields of view of 6.6 cm and 13 cm were acquired. The signal was quantified in photon flux per second (p s^−1^) using the Living Image Software (version 4.7.3.20616, Perkin-Elmer) by selecting a rectangular region of interest (ROI) placed over the catheters or organs.

### In vivo antibiofilm efficacy study

Mature 24 h-old in vivo biofilms were treated for 7 days with nanosystems or free compounds. Functionalized CAS-loaded LNPs and free CAS were administered intraperitoneally once a day for 7 days at a CAS dose of 4 mg kg^−1^ of body weight. The volume of the intraperitoneal injection per mouse was 250 µL. The functionalized MOX-loaded MLNs and free MOX were administered intraperitoneally twice a day for 7 days at a MOX dose of 5 mg kg^−1^ of body weight. Uninfected and untreated infected control groups were treated twice a day for 7 days with sterile type I water by intraperitoneal injection. Mice were imaged on days 1, 2, 5, 7, and 8 after implantation. On day 1 after implantation, the mice were imaged before the treatment. Anesthetized animals were placed in the In Vivo Imaging System (IVIS Spectrum, Perkin-Elmer, Waltham, Massachusetts, USA). Frames were acquired with a field of view of 23 cm. Bioluminescence imaging was acquired using 40 s exposure time, binning 2, f/stop = 1. After the treatment, the animals were anesthetized with a mixture of isoflurane in oxygen (1.5–2%, v/v) and sacrificed by cervical dislocation. The catheters were harvested and imaged using frames with a field of view of 13 cm. The signal was quantified in photon flux per second (p s^−1^) using the Living Image Software (version 4.7.3.20616, Perkin-Elmer) by selecting a ROI placed over the catheters. Afterward, the catheters were washed twice with PBS 1x and sonicated at room temperature for 10 min in 200 µL of PBS 1x. Spleen, kidneys, and liver were also harvested and further submerged in 500 µL of PBS 1x and homogenized for 40 s (FastPrep-24^TM^; MP Biomedicals, LLC; Santa Ana, California, USA). Then, the samples were serially diluted in PBS 1x and spot-plated (10 µL) on TSA plates. The plates were incubated overnight at 37 °C to allow CFUs counting.

### Statistical analysis

Statistical analysis was performed using Graphpad Prism Software (version 7.03; IBM, Armonk, New York, USA). All in vitro assays were performed at least three independent times. For in vivo assays, at least three biological replicates were used.

### Reporting summary

Further information on research design is available in the Nature Research Reporting Summary linked to this article.

## Supplementary information

Supplementary information Pinto_2023

Reporting Summary

## Data Availability

All data generated or analyzed during this study are included in this published article and in Supplementary information.
