# Supplementary material for: Non disseminative nano-strategy against in vivo Staphylococcus aureus biofilms
Source: NPJ Biofilms Microbiomes. 2023 Jun 17;9:39. doi: 10.1038/s41522-023-00405-4 (PMC10275942; doi:10.1038/s41522-023-00405-4)
Supplement: Supplementary file 1 — Supplementary information Pinto_2023 [file 41522_2023_405_MOESM1_ESM.pdf]

Supplementary Information

Non disseminative nano-strategy against *in vivo Staphylococcus aureus* biofilms

Rita M. Pinto[1,2], Saleh Yazdani[2], Catarina Leal Seabra[1], Martine De Jonge[2], Mukaddes Izci[3], Rebeca Cruz[1], Susana Casal[1], Stefaan J. Soenen[3], Salette Reis[1], Cláudia Nunes[1*], Patrick Van Dijck[2,*]

[1] LAQV, REQUIMTE, Departamento de Ciências Químicas, Faculdade de Farmácia, Universidade do Porto, 4050-313 Porto, Portugal

[2] Laboratory of Molecular Cell Biology, Institute of Botany and Microbiology, KU Leuven, 3001 Leuven, Belgium

[3] NanoHealth and Optical Imaging Group, Translational Cell and Tissue Research Unit, Department of Imaging and Pathology, KU Leuven, Leuven, Belgium

*Corresponding authors:

Patrick Van Dijck: patrick.vandijck@kuleuven.be

Cláudia Nunes: cdnunes@ff.up.pt

1. *In vitro* drug release study

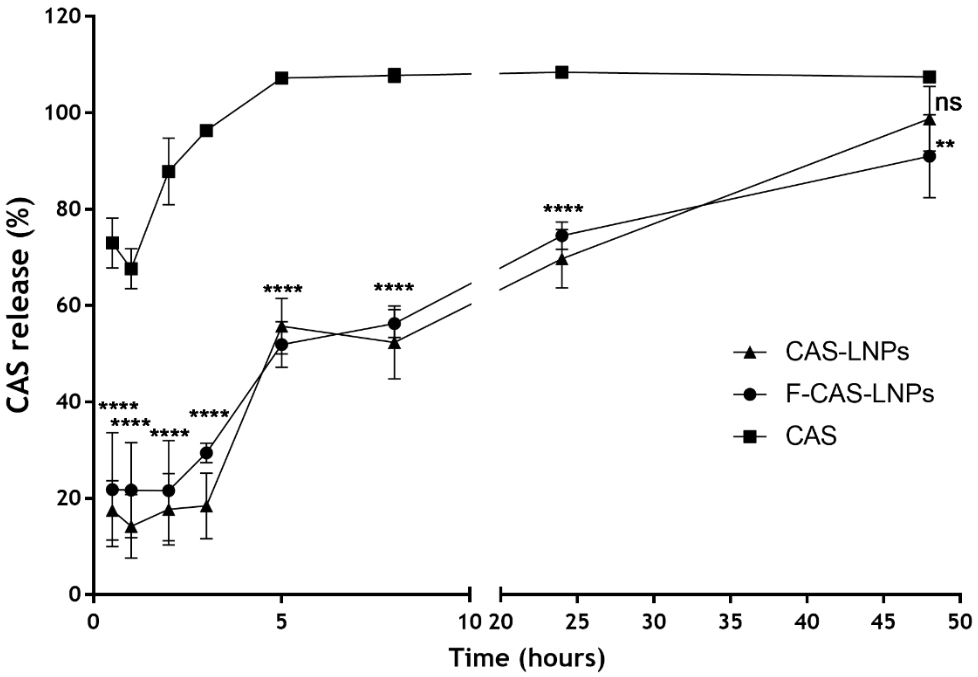

Supplementary Figure 1. ***In vitro*** **CAS release profiles from the CAS-loaded LNPs (CAS-LNPs and F-CAS-LNPs) and the free CAS (CAS) in pH 7.4 during 48h, using the dialysis diffusion technique.** Values represent the mean ± SD of three independently produced formulations.

2. *In vitro* biocompatibility studies: hemolysis assay

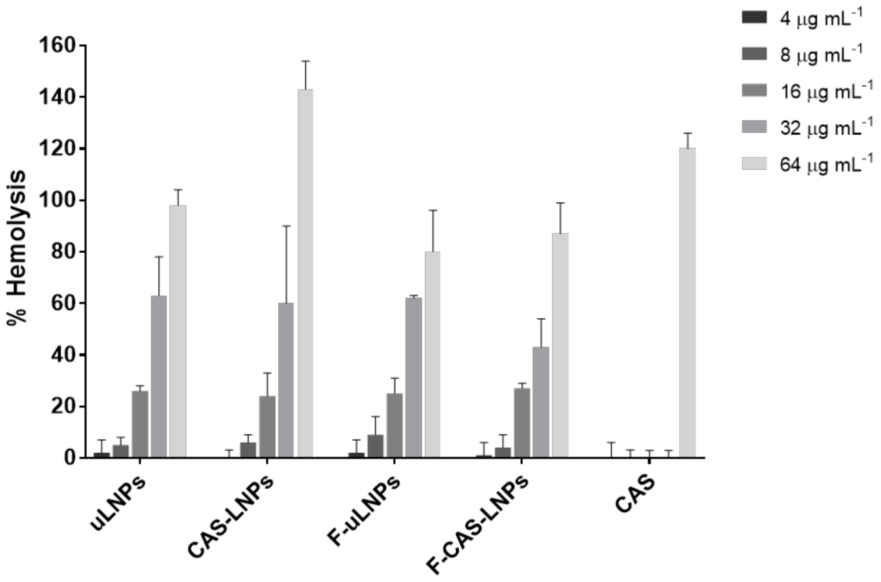

Supplementary Figure 2. Evaluation of the hemolytic activity of the developed LNPs and free CAS. The LNPs and free compound at the CAS concentrations 4, 8, 16, 32, and 64 µg mL^{-1} were tested. For positive and negative controls, saline solution (0.85%, w/v) and Triton^{TM}-100x (1%, v/v) were used, respectively.

3. *In vitro* antibiofilm activity: biofilm biomass study

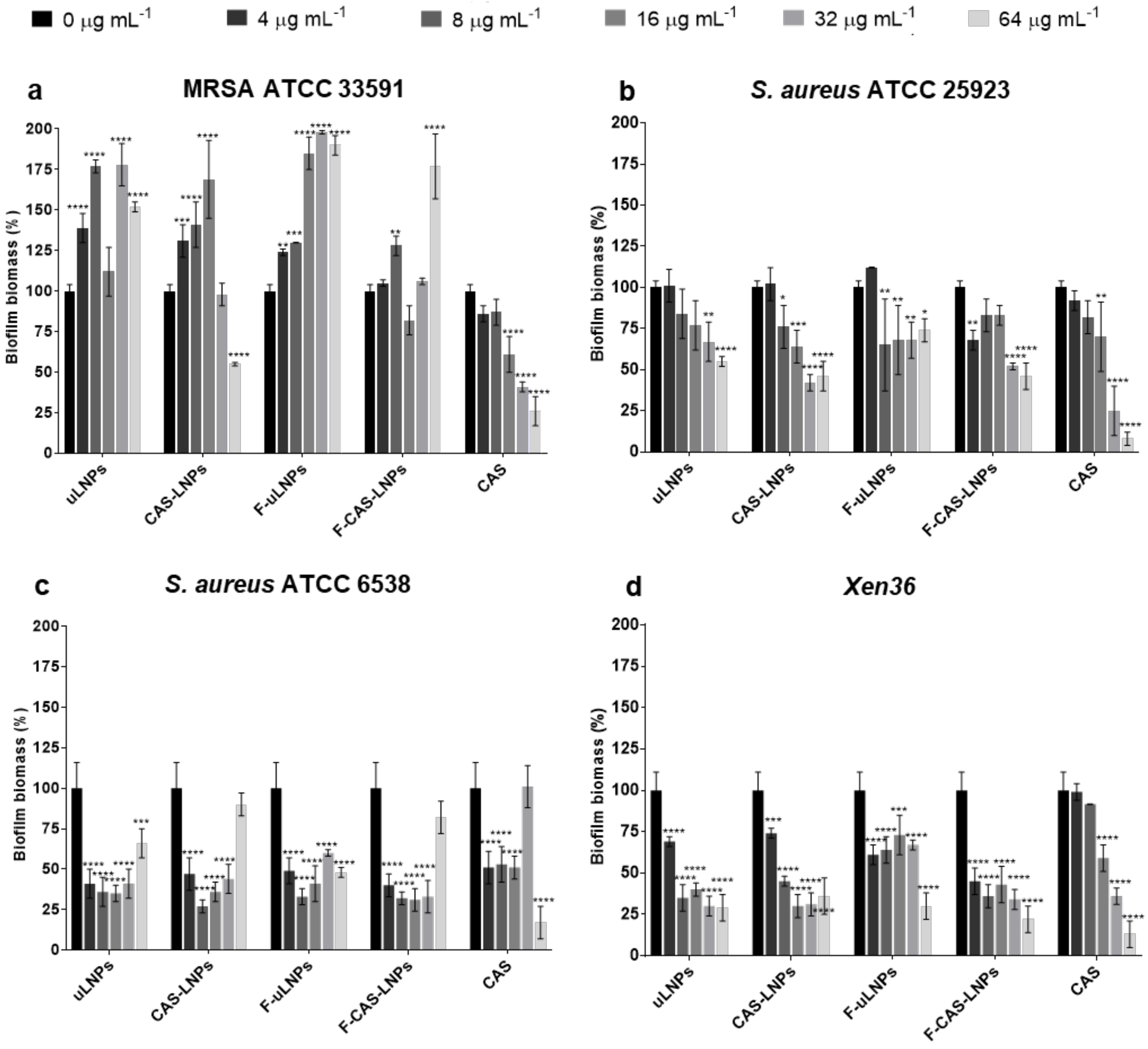

Supplementary Figure 3. Quantification of biofilm biomass (crystal violet staining) after a 24h treatment with LNPs or free CAS at the CAS concentrations of 0, 4, 8, 16, 32 and 64 μg mL^{-1}. Prior to the treatment, the biofilms of **a** MRSA ATCC 33591, **b** *S. aureus* ATCC 25923, **c** *S. aureus* ATCC 6538, and **d** the bioluminescent strain *Xen36* were grown in 96-well plates for 24h. Untreated biofilms (0 μg mL^{-1}) were used as a positive control. The values are represented as the mean ± SD. *p<0.05, **p<0.01, ***p<0.001, ****p<0.0001 relatively to 0 μg mL^{-1}. Statistical analysis: two-way ANOVA, Dunnett's multiple comparisons test.

4. *In vivo* biodistribution study

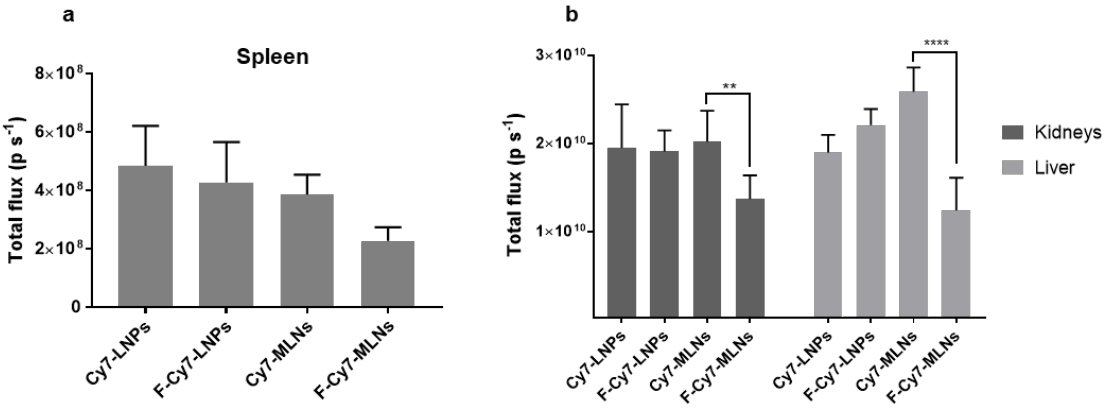

Supplementary Figure 4. *In vivo* biodistribution study. Fluorescence signal intensity of **a** spleen and **b** kidneys and liver. The organs were acquired *ex vivo* at 24h post-administration of the Cy7-loaded formulations. Fluorescent signals are expressed in photons per second (p s^{-1}). The values are represented as the mean ± SD for three animals (each implanted with four catheters). **p<0.001, ****p<0.0001. Statistical analysis: two-way ANOVA, Tukey's multiple comparisons test.

5. *In vivo* antibiofilm efficacy study

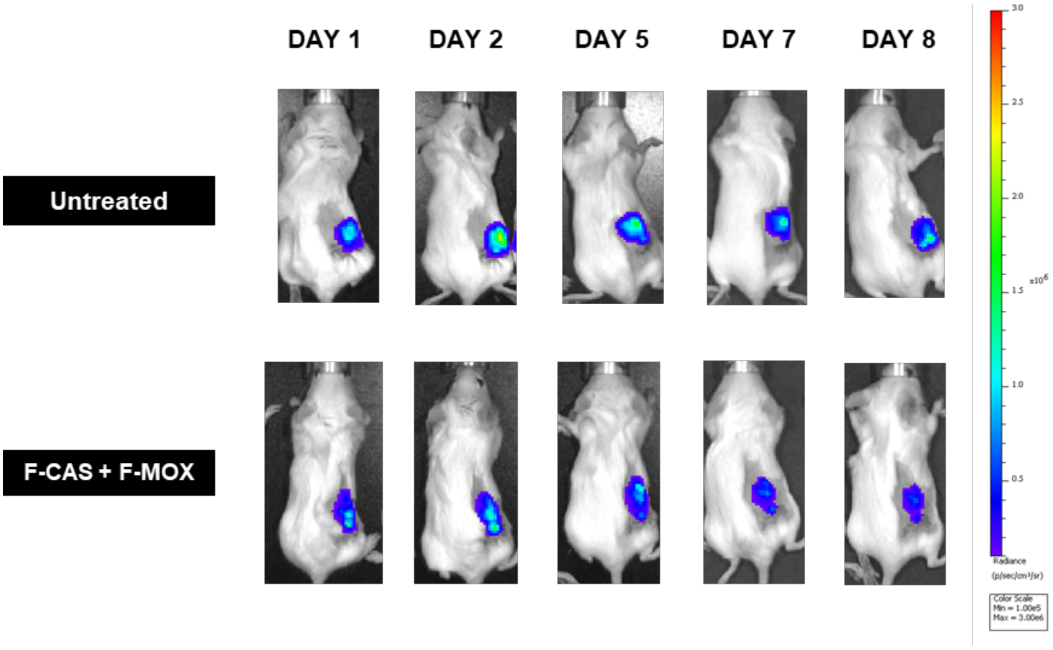

Supplementary Figure 5. *In vivo* antibiofilm efficacy study. Representative bioluminescence images of *Xen36* biofilms at different time-points (1, 2, 5, 7, and 8 days after implantation) for the groups untreated and treated with the combination of F-CAS-LNPs with F-MOX-MLNs. On day 1, mice were imaged before the treatment.

6. CAS calibration curve

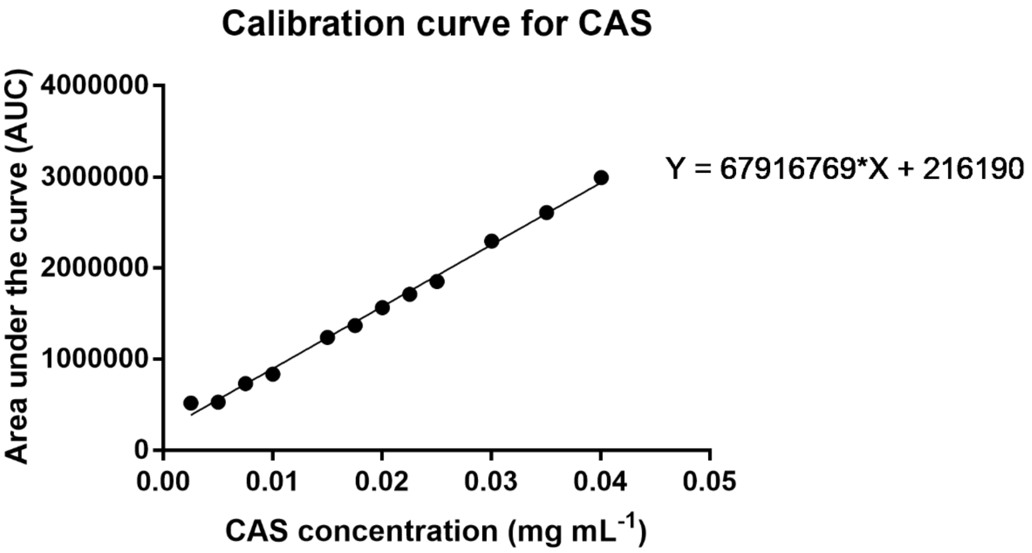

Supplementary Figure 6. High-performance liquid chromatography calibration curve for CAS.
